# How Does Bio-Organic Fertilizer Combined with Biochar Affect Chinese Small Cabbage’s Growth and Quality on Newly Reclaimed Land?

**DOI:** 10.3390/plants13050598

**Published:** 2024-02-22

**Authors:** Juan Wang, Biyu Zhai, Danyi Shi, Anquan Chen, Chuncheng Liu

**Affiliations:** 1 College of Hydraulic Science and Engineering, Yangzhou University, Yangzhou 225009, China; mz120221136@stu.yzu.edu.cn (B.Z.); mz120211074@stu.yzu.edu.cn (D.S.); mx120200610@yzu.edu.cn (A.C.); 2 Institute of Farmland Irrigation of CAAS, Xinxiang 453002, China; 3 China Shangqiu Station of National Field Agro-Ecosystem Experimental Network/National Agricultural Experimental Station for Agricultural Environment, Shangqiu 476000, China

**Keywords:** biochar, Chinese small cabbage, water productivity, plant growth, photosynthetic characteristics, fertilizer reduction

## Abstract

The cultivated land area in China is approaching the red line for farmland protection. Newly reclaimed land possesses a large exploratory potential to become a reserved land resource. Identifying a fertilization strategy is vital for improving the poor properties and weak fertility of newly reclaimed land. An experiment was conducted to study the effects of traditional compound fertilizer (Fc) or bio-organic fertilizer (Ft), alone or in combination with biochar addition (6.85 t·ha^−1^ and 13.7 t·ha^−1^) on the growth, photosynthesis, yield and quality of Chinese small cabbage (CSC) plant. The results showed that compared to single compound fertilizer application, bio-organic fertilizer application promoted the plant’s growth, indicated by the plant height, stem diameter and leaf area index (LAI), and significantly enhanced the yield and dry matter accumulation of CSC. In terms of the combination with biochar, the promoting effects were positively related to the biochar addition rate in the compound fertilizer group, while it was better to apply bio-organic fertilizer alone or in combination with biochar at a low rate of 6.85 t·ha^−1^. The highest yield was obtained under B2Fc and B1Ft with 29.41 and 37.93 t·ha^−1^, respectively, and the yield under B1Ft was significantly higher than that under B2Fc. The water productivity (WP) significantly improved in response to both kinds of fertilizer combined with biochar at 6.85 t·ha^−1^. There was a significant difference between the photosynthetic characteristics of plants treated with single-compound fertilizer and those treated with bio-organic fertilizer. The photosynthetic characteristics increased under compound fertilizer combined with biochar, while they regressed under bio-organic fertilizer combined with biochar. The quality of CSC, especially that of soluble sugars and total phenolics, improved under single bio-organic fertilizer application compared with that under single-compound fertilizer. The nitrite content of the plants increased with increasing biochar addition rate in both fertilizer groups. In conclusion, there is a significant promoting effect of applying bio-organic fertilizer to replace chemical fertilizer alone or combining compound fertilizer with low-rate biochar addition on newly reclaimed land. It is a recommended fertilization strategy to substitute or partially substitute chemical fertilizer with bio-organic fertilizer combined with biochar in newly reclaimed land, and it is of great significance to achieve fertilizer reduction.

## 1. Introduction

According to the third National Land Survey, the cultivated land in China decreased by 7.33 million ha by the end of 2019. There was an average annual decrease of more than 0.67 million ha since the end of 2009, and the cultivated land area was approaching the red line of farmland protection at 120 million ha [1]. Newly reclaimed land, as a vital reserved land resource, has great productive potential to be explored. However, newly reclaimed soil usually has low productivity, poor properties and weak fertility. Therefore, it is of great significance to quickly promote fertility of reclaimed soil by rational fertilization management, which is highly scientifically important for realizing the efficient development and utilization of reserved land.

At present, the compound fertilizer is a kind of chemical fertilizer commonly used to increase crop yield. Excessive application of chemical fertilizer is becoming common in pursuit of high yields. However, excessive fertilization leads to a decrease in fertilizer utilization during the season and easily induces damage to the soil ecological balance and microbial community, which is not conducive to soil structural stability or land productivity [2]. To explore rational fertilization management, previous scholars have studied the effects of bio-organic fertilizer or its combination with other fertilizer on soil and crops [3,4]. Wang et al. [5] reported that the yield and quality of dryland wheat improved in response to bio-organic fertilizer application and its application rate. It is reported that bio-fertilizer increased soil nutrients through natural processes of nitrogen fixation, phosphorus dissolution and biodegradation, then promoted plant growth and was friendly to the ecological environment [6]. In organic-fertilizer-treated soils, more key taxa, such as Amycolatopsis, Variovorax and Gemmatimonas, were positively associated with soil nutrient concentrations and crop yield [7]. Wang et al. [8] revealed the promotional effect of bio-organic fertilizer on the soil microbial community as well as Chrysanthemum’s growth and yield in a continuous monoculture system. In general, bio-organic fertilizer has positive effects on soil improvement and plant growth as well as yield. However, most of the previous studies have focused mainly on adding bio-organic fertilizer to existing fertilization schedules, or comparing the effects of bio-organic fertilizer and no fertilization; it is not clear whether bio-organic fertilizer can replace chemical fertilizer or fundamentally achieve fertilizer reduction.

Biochar is a new type of soil amendment, known as “black gold”, and is used by international academics. Many researchers have shown that biochar, as an organic amendment, has significant advantages in terms of improving soil structure, increasing soil nutrients, and ensuring better quality and increasing plant yields when added to soil [9,10]. Xu [11] reported that a biochar addition rate of 3 t·ha^−1^ ensured stable maize yields when fertilizer application was reduced by 30%, and 3, 5 and 7 t·ha^−1^ biochar significantly increased maize yields at adequate nutrient levels. Moreover, combining biochar with fertilization has been reported to be an efficient way to improve land productivity [12,13]. Zhang et al. [14] noted that applying fertilizer in combination with biochar or applying carbon-based fertilizer to farmlands can increase the soil phosphorus content and effectively prevent nitrate nitrogen from moving to deep soil. Si’s [15] research showed that, compared to traditional fertilization methods, combining biochar with fertilizer could increase paddy yield to a certain extent; in terms of nitrogen absorption, yield firstly increased and then decreased with increasing biochar addition. Overall, combining biochar with fertilizer seems to be a feasible way to enhance plant growth, improve fertilizer utilization efficiency and possibly achieve chemical fertilizer reduction.

Based on the above, we hypothesize that bio-organic fertilizer mainly made from harmless treatment products of animal carcasses or bio-fertilizer in combination with biochar can substitute or partially substitute for chemical fertilizer to maintain the same plant yield and quality. We investigated the effects of fertilization strategies (a single application of traditional compound fertilizer or bio-organic fertilizer, as well as the combination of two fertilizers combined with biochar addition at low and high rates) on the growth, photosynthesis, yield and quality of Chinese small cabbage. The aims of this work are (1) to investigate the impact of traditional compound fertilizer and bio-organic fertilizer combined with biochar addition and its combined effects on Chinese small cabbage’s growth, photosynthetic characteristics, yield and quality; (2) to discuss the possibility of bio-organic fertilizer substituting compound fertilizer; and (3) to propose a fertilization strategy that can effectively improve the productivity of newly reclaimed fields and crop quality, thereby increasing cultivated land resources and guaranteeing the sustainable development of agriculture.

## 2. Materials and Methods

### 2.1. Tested Soil, Biochar and Bio-Organic Fertilizer Prepartion

The tested soil was collected from the Agricultural Soil and Water Environment and Ecology experimental site at Yangzhou University. As a new experimental site, the former soil was uncultivated land with low productivity and was defined as reclaimed land. The soil was collected from the top 0–25 cm in a “*Z*” shape. The large gravel was removed, and the soil was air-dried and subsequently sieved through a 2 mm sieve. The soil particle size composition was measured using a Malvern laser particle size analyzer (MS–3000). The proportions of clay (particle size < 0.002 mm), silt (particle size 0.002–0.02 mm), and sand (particle size 0.02–2 mm) were 4.38%, 32.76%, and 62.86%, respectively. The test soil texture can be classified as a sandy loam according to the International Soil Classification System [16]. The soil bulk density was 1.37 g·cm^−3^, the soil available nitrogen content was 32.08 mg·kg^−1^, the soil organic matter content was 7.20 g·kg^−1^, the saturated water content was 38.05%, the electronic conductivity was 0.17 mS·cm^−1^, and the saturated hydraulic conductivity was 8.07 × 10^−4^ cm·s^−1^. 

The compound fertilizer used in the study was produced from Stanley Fertilizer Corporation. The total nutrient content of the fertilizer was ≥45%, and percentages of the N-P-K ratio were 15:15:15. The bio-organic fertilizer mainly originates from the dry residue of harmless disposal of livestock and poultry carcasses. The harmless disposal method commonly known as the chemical preparation process is a safe and efficient way to treat dead livestock and poultry carcasses. In the process of harmless disposal, first, the carcasses of livestock and poultry were dismembered and placed in containers at a high temperature (over 140 °C), and a high pressure (over 0.5 MPa), saw power was added during this process. After 4 h of continuous action, the dead animal carcass is dissolved and transformed into sterile solution and dry matter from the bone residue, after which all the pathogenic microorganisms are killed [17,18]. The dry residue was composted by adding microbial bacteria agent and then dried to obtain bio-organic fertilizer. The mass fractions for the nitrogen and carbon contents of the bio-organic fertilizer were 7.28% and 50.91%, respectively. The biochar used in this study was bought from Henan Lize Environmental Protection Technology Co. Ltd. (Zhengzhou, China), which originated from corn straw, and pyrolyzed at 600 °C for 12 h, with size of 0.15 mm, pH of 9.0 and ash content of 10%; its organic carbon content was 40%.

The pots were plastic buckets with a height of 25 cm and a diameter of 23 cm, and small holes were drilled at the bottom for free drainage. To prevent the upper fine particles from plugging the apertures, 2 layers of geotextile were set at the bottom before packing the soil. Prior to the experiment, the collected soil was air-dried and sieved via a 2 mm stainless steel sieve. The amounts of biochar, fertilizer and soil were calculated, weighed and mixed thoroughly to minimize variability before the pots were filled. The soil was packed into pots by every 5 cm layer to achieve a bulk density of approximately 1.37 g·cm^−3^. The total soil depth of the pot was 20 cm, with a 5 cm gap for retaining water. Each layer was lightly raked before the next layer was packed to minimize discontinuities between layers.

Prior to the experiment, the pots were irrigated with 860 mL of distilled water, according to the saturated water content of the soil. Nine sowing locations with a width of 5 cm were selected in each pot, and three plant seeds were sowed on each spot. Chinese small cabbage (CSC) was sown on 17 May 2022, and harvested on 30 June 2022, for a total growth period of 45 days. The seedlings were thinned after emergence, and only one plant seedling was kept at each sowing location. During the trial, the pots were irrigated every 2–3 days to maintain suitable soil moisture content (80% of field capacity), and natural rainfall was avoided during the growth period. The pots were weighed by an electronic scale (the accuracy was 0.1 g), and the difference in weight was the amount of water required for irrigation.

The experiment was carried out in a greenhouse at the South Road Campus of Yangzhou University (119°25′17″ E, 32°22′33″ N) in southern China from May to June 2023. The site is classified as having a humid subtropical climate (Köppen climate), which is a typical climate in eastern China. The daily temperature inside the greenhouse was in the range of 25–30 °C, and the humidity was around 50–55%.

### 2.2. Experimental Site and Design

This study evaluated six treatments with three replicates. Three biochar addition rates and two fertilization types were used in the experiment. Based on the economic considerations from field practice, the amount of biochar applied in pot trials should be low, not exceeding 20 t·ha^−1^. Therefore, 0.25% and 0.5% of the soil mass in the 20 cm topsoil layer per hectare (to simulate 6.85 t·ha^−1^ and 13.7 t·ha^−1^) were selected as biochar addition rates in this trial, and no addition was used as a control. Two fertilizer types, conventional compound fertilizer (Fc) and bio-organic fertilizer (Ft), were used, and the application rate was equivalent to a nitrogen application of 150 mg·kg^−1^. The combination of Fc application without biochar addition (B0Fc) was used as a CK treatment to simulate local conventional cultivation. The detailed experimental treatment schemes are shown in Table 1.

### 2.3. Observation and Measurement

At the beginning of the experiment, 27 Chinese small cabbage seeds were meticulously sown in each pot, and the emergence rate was recorded on the 10th day. During the growth stage, a steel tap (with an accuracy of 0.01 m) was used to measure the plant height from the ground to the highest point of a natural extension of all the leaves, and the stem diameter was measured by a digital Vernier caliper with an accuracy of 0.01 m.

The leaf area index (LAI) was calculated as follows by measuring the area of a leaf on the 20th, 27th, 31st, 40th and 45th days after sowing:

(1) $$LAI=\frac{\sum_{i=1}^{n}A_{i}}{A}$$

where *A_i_* is the area of leaf (cm^2^), calculated with planimeter by copy the leaf on paper; *A* is the pot area (cm^2^).

The Chinese small cabbage was carefully removed from the soil at physiological maturity, the remaining soil of the roots was rinsed off, any excess rinsing moisture was removed and the fresh weight of each plant was determined. We measured the fresh weight of the whole CSC plant population in each pot to obtain the harvest yield per pot and subsequently converted the yield into the CSC yield in kg·ha^−1^ by using the pot area. The roots were separated and weighed to determine the fresh weight; the remaining part of the plant was weighed to determine the aboveground fresh weight. Dry matter was measured by drying the aboveground part and the root part at 105 °C for 30 min and then oven drying at 75 °C until a constant weight was reached.

The SPAD value was measured by a SPAD-502PLUS chlorophyll meter (KONICA MINOLTA, Tokyo, Japan); 3 plants were selected for each pot, and the average value of all the leaves was recorded as the SPAD value for each pot. The photosynthetic characteristics were measured by an LCpro-SD fully automated portable photosynthesis analyzer (Analytical Development Co., Ltd., Hoddesdon, UK) on a sunny day prior to harvest. At four specific time points (8:00 a.m., 11:00 a.m., 2:00 p.m., and 5:00 p.m.), three moderately developed Chinese small cabbage plants were deliberately selected from each pot to perform the photosynthesis measurements meticulously.

The total water consumption (*ET*) was calculated as follows, based on the irrigation water amount:

(2) ET=P+I+ΔW−R−D

where *P* is the precipitation, *I* is the cumulative irrigation water amount (mm), ΔW is the soil moisture variation (mm), *R* is the surface runoff (mm), and *D* is the deep percolation (mm) [19].

The whole growth period of Chinese small cabbage was sheltered from rain, and no surface runoff was generated and no leachate occurred, so the equation can be simplified as follows:

(3) ET=I+ΔW−D

Thus, the water productivity (*WP*) was calculated as follows:

(4) $$WP=\frac{Y}{ET}$$

where *Y* is the yield of Chinese small cabbage (kg·ha^−1^).

The soluble protein content of plants was determined by Coomassie brilliant blue colorimetry, the soluble sugar content was determined by anthrone colorimetry, the total phenol content was measured by Folin phenol method, and the nitrite content was determined by spectrophotometry. 

### 2.4. Data Analysis and Statistics

Microsoft Excel 2010 (Microsoft Corporation, Redmond, WA, USA, 2010) was used for data calculation and processing. The significant differences among treatments were determined by Duncan’s test, using the SPSS 20.0 statistical program (International Business Machines Corporation, Armonk, NY, USA, 2011). Duncan’s method in two-way ANOVA was used to test the significance difference between the values at different levels of 95% and 99%. The figures were plotted and exported by Origin 8.0 (OriginLab Corporation, Northampton, MA, USA, 2007).

## 3. Results

### 3.1. Growth of Chinese Small Cabbage

**Figure 1:**
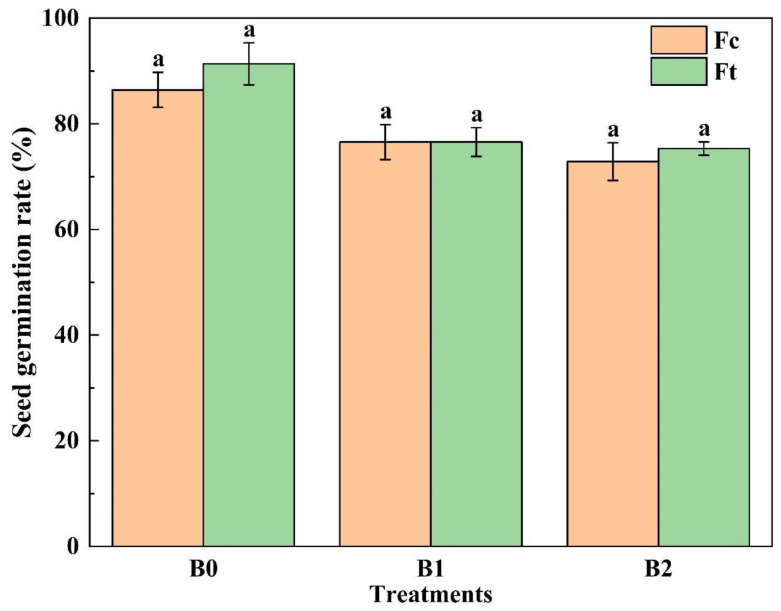
The seed germination under treatments. Notes: The same lowercase letters indicate no significant difference (at *p* > 0.05) according to Duncan’s test. B0, B1 and B2 represent the column group of biochar addition rates of 0, 6.85, and 13.7 t·ha^−1^, respectively; with the addition of compound fertilizer (yellow columns) or bio-organic fertilizer (green columns), the same as below.

The growth of Chinese small cabbage plants was evaluated by seed germination, plant height, stem diameter and leaf area index (LAI). Figure 1 shows that more than 70% of the seeds germinated under all the treatments, and there was no significant difference between them (*p* > 0.05). Seed germination was greatest (91.36%) under B0Ft treatment, which was 5.7% greater than that under B0Fc treatment.

**Figure 2:**
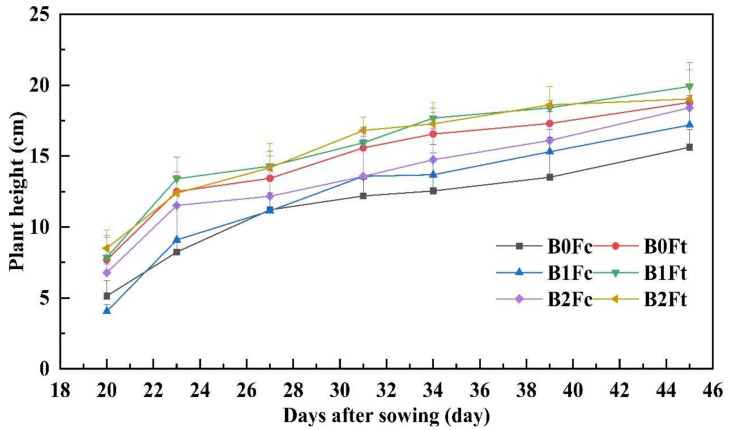
The plant height variation with days after sowing. Notes: “B” represents biochar; B0, B1 and B2 represent the biochar addition rates of 0, 6.85 and 13.7 t·ha^−1^, respectively; “F” represents fertilizer; Fc and Ft represent the compound fertilizer and bio-organic fertilizer, respectively. For example, “B1Ft” is the treatment of biochar addition at 6.85 t·ha^−1^ combining the bio-organic fertilizer application.

**Figure 3:**
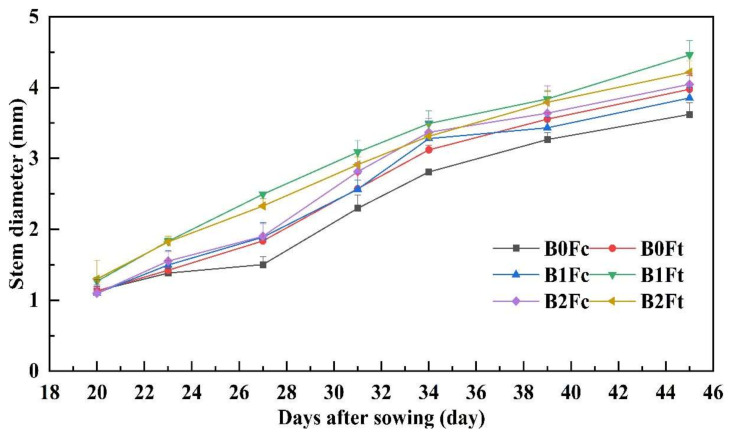
The variation in stem diameter with days after sowing. Notes: “B” represents biochar; B0, B1 and B2 represent the biochar addition rates of 0, 6.85 and 13.7 t·ha^−1^, respectively; “F” represents fertilizer; Fc and Ft represent the compound fertilizer and bio-organic fertilizer, respectively. For example, “B1Ft” is the treatment of biochar addition at 6.85 t·ha^−1^ combining the bio-organic fertilizer application.

Plant height and stem diameter were monitored beginning 20 days after sowing; their dynamics are shown in Figure 2 and Figure 3. Figure 2 shows that the plant height quickly increased in the first 23 days and then slowly increased to a stable value. The plant height under the three treatments with bio-organic fertilizer (Ft) was always greater than that treated with compound fertilizer (Fc) treatments. In addition, plant height was increased with increasing biochar addition rate within each fertilizer type group. Almost 27 days after sowing, the plant height in all treatments was greater than that under B0Fc; the other treatments became more pronounced over time. At harvest, the heights of other plants were only almost 10.1–27.5% greater than that of B0Fc plants, which was only 15.62 cm.

The stem diameter steadily increased with sowing days after germination. According to the results of plant height, Figure 3 shows that the stem diameter under B0Fc was also much lower than others. Under the same biochar addition, the stem diameter with bio-organic fertilizer was greater than that under compound fertilizer. In the common fertilizer group, the stem diameter increased with increasing biochar addition, while the stem diameter was greatest with medium biochar addition (B1Ft) in the bio-organic fertilizer group.

**Figure 4:**
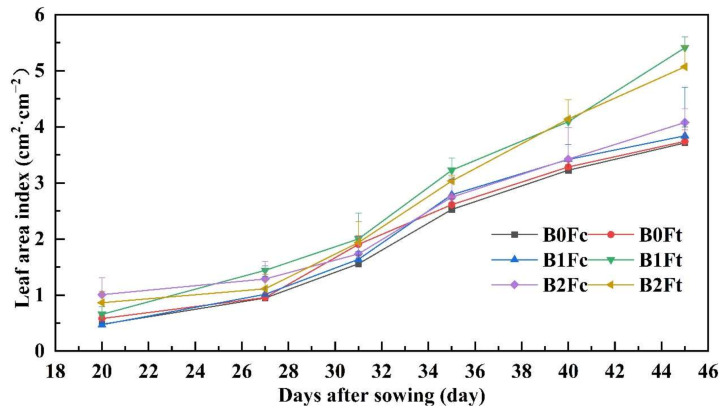
The LAI variation with days after sowing. Notes: “B” represents biochar; B0, B1 and B2 represent the biochar addition rates of 0, 6.85 and 13.7 t·ha^−1^, respectively; “F” represents fertilizer; Fc and Ft represent the compound fertilizer and bio-organic fertilizer, respectively. For example, “B1Ft” is the treatment of biochar addition at 6.85 t·ha^−1^ combining the bio-organic fertilizer application.

Figure 4 shows that the leaf area index (LAI) slowly increased before 27 days after sowing and then rapidly increased to a peak at harvest. Approximately 20 days after sowing, the LAI under the high biochar addition rate for B2Fc and B2Ft was greater than that under the other treatments. At harvest, the LAI under B1Ft and B2Ft was greater than the others, which indicated that biochar promoted the early growth of CSCs and that bio-organic fertilizer mainly promoted plant growth in the later stage. The LAI under treatment with compound fertilizer without biochar addition (B0Fc) was always at the minimum, as was the plant height and stem diameter tendency, which indicates that both biochar and bio-organic fertilizer were beneficial for improving CSC growth.

### 3.2. Dry Matter, Yield and Water Productivity

**Figure 5:**
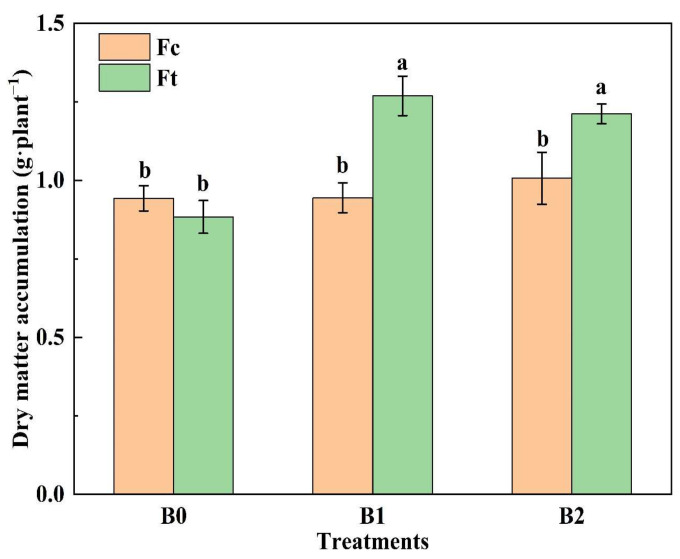
The dry matter accumulation under the treatments. Notes: “B” represents biochar; B0, B1 and B2 represent the biochar addition rates of 0, 6.85 and 13.7 t·ha^−1^, respectively, with the addition of compound fertilizer (yellow columns) or bio-organic fertilizer (green columns). The different lowercase letters indicate significant difference (at *p* < 0.05) according to Duncan’s test.

Figure 5 shows that that the accumulation of dry matter at harvest under B1Ft and B2Ft was significantly greater than others (*p* < 0.05), while there was no significant difference between the rest treatments. Notably, the dry matter content under B0Ft was the lowest, and was even lower than that under B0Fc; subsequently, the dry matter content dramatically increased to its highest value under B1Ft, an increase of 44.20%. Taken together, these findings indicate that biochar can stimulate the release of nutrients from bio-organic fertilizer, thus enhancing plant dry matter accumulation.

**Figure 6:**
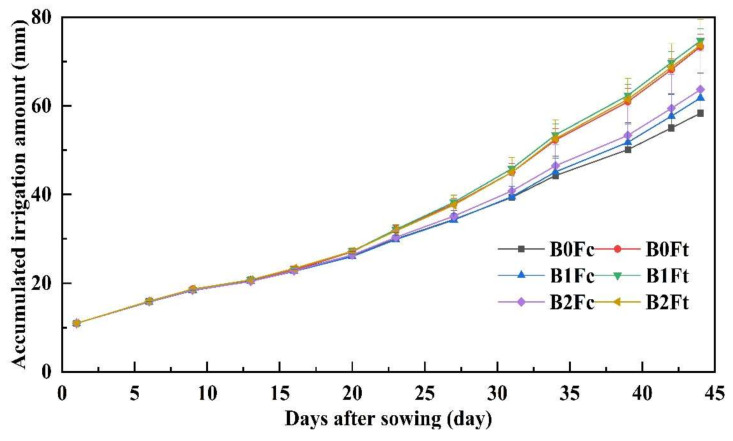
The accumulated irrigation amount during plant growth under treatments. Notes: “B” represents biochar; B0, B1 and B2 represent the biochar addition rates of 0, 6.85 and 13.7 t·ha^−1^, respectively. “F” represents fertilizer; Fc and Ft represent the compound fertilizer and bio-organic fertilizer, respectively. For example, “B1Ft” is the treatment of biochar addition at 6.85 t·ha^−1^ combining the bio-organic fertilizer application.

The accumulated irrigation amount is shown in Figure 6 and reflects water consumption, including plant transpiration and soil evaporation. The curves almost coincided before the first 20 days after sowing, and the difference gap between irrigation amount of the compound fertilizer group and that of the bio-organic fertilizer group becomes increasingly larger.

The total irrigation water amount, ET, Chinese small cabbage yield and WP were calculated and are shown in Table 2. As shown in Table 2. the total irrigation water amount under B1Ft treatment was significantly greater than that under compound fertilizer treatment group. The ET under the bio-organic fertilizer treatments was significantly greater than that under the compound fertilizer without biochar addition (B0Fc), while there was no significant difference among the treatments inside each group. The yield was significantly lower under B0Fc and B0Ft (treatments without biochar addition); in the compound fertilizer group, a greater yield was obtained under B2Fc with highest biochar addition. However, in bio-organic fertilizer group, the highest yield was obtained under B1Ft of 37.93 ± 4.46 t·ha^−1^, which indicated that a biochar addition threshold should be met when combined with bio-organic fertilizer. The water productivity (WP) was also significantly lower under B0Fc and B0Ft, which indicated that biochar addition can enhance yield and WP. Interestingly, the highest WPs in the compound fertilizer and bio-organic fertilizer groups were obtained from B1Fc and B1Ft (medium biochar addition rate combined with fertilizer), with 20.51 kg·m^−3^ and 26.67 kg·m^−3^, respectively.

### 3.3. Photosynthetic Characteristics

**Figure 7:**
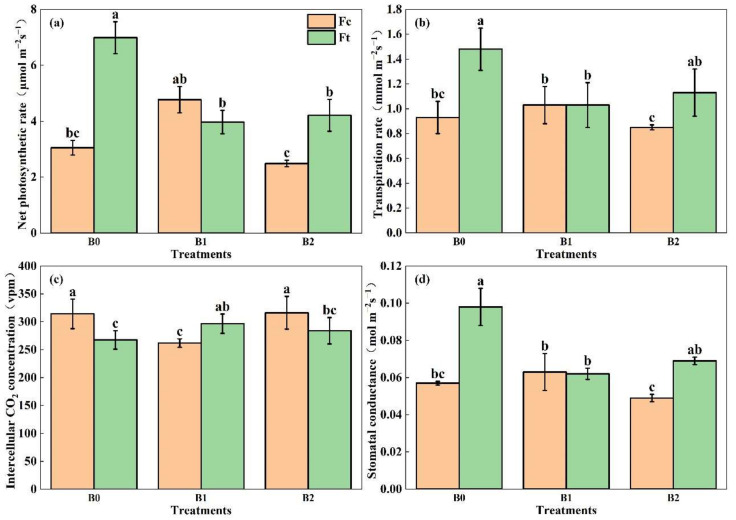
The net photosynthetic rate (**a**), transpiration rate (**b**), intercellular CO_2_ concentration (**c**) and stomatal conductance (**d**) of Chinese samll cabbage under the different treatments. Notes: “B” represents biochar; B0, B1 and B2 represent the biochar addition rates of 0, 6.85 and 13.7 t·ha^−1^, respectively, with the addition of compound fertilizer (yellow columns) or bio-organic fertilizer (green columns). The different lowercase letters indicate significant difference (at *p* < 0.05) according to Duncan’s test.

The photosynthetic characteristics of Chinese small cabbage plants are shown in Figure 7. The net photosynthetic rate reflects the speed of nutrient accumulation in plants. The results in Figure 7a indicate that the net photosynthetic rate of B0Ft was significantly greater than others. In the common group, the net photosynthetic rate was highest under B1Fc and significantly greater than that under B2Fc, which indicates that biochar addition can improve the plant net photosynthetic rate, while the rate should not be excessive. As the amount of added biochar increased, the net photosynthetic rate initially decreased and then slightly increased in the bio-organic fertilizer group. Compared to B0Ft, biochar significantly reduced the net photosynthetic rate, with a decrease ranging from 40.0% to 43.2%, which indicated that biochar led to a reduction in the net photosynthetic rate in response to bio-organic fertilizer.

The transpiration rate of Chinese small cabbage reflects its water demand and consumption, making it a crucial indicator for assessing plant water requirements. The maximum transpiration rate was 1.481 mmol·m^−2^s^−1^ under B0Ft, which was significantly greater than that under the other treatments except for under B2Ft. The transpiration rates of B0Ft and B2Ft, which were treated with bio-organic fertilizer without biochar or with a high addition rate of biochar, were significantly greater than those under B0Fc and B2Fc. In the compound fertilizer group, there was a slight difference among the three treatments, which indicated that biochar addition had little effect on the plant transpiration rate when combined with compound fertilizer. In the bio-organic fertilizer group, the transpiration rate initially decreased and then slightly increased with increasing addition of biochar. Compared to B0Ft, biochar reduced the transpiration rate by 30.5% and 23.5% under B1Fc and B2Fc, respectively. Moreover, biochar reduced the transpiration rate, with a greater impact observed at low application rates when bio-organic fertilizer was applied. A comparison of the two fertilizer groups revealed that the average transpiration rate under bio-organic fertilizer was 29.6% greater than that under common fertilizer, which indicates that bio-fertilizer enhanced plant transpiration. 

The intercellular CO_2_ concentration ranged from 262 to 316.22 vpm. Compared to single compound fertilizer application, the intercellular CO_2_ concentration decreased signiciantly under B1Fc and then increased to 316.22 vpm under B2Fc, while there is no signiciant difference with B0Fc. Conversely, the intercellular CO_2_ concentration under B1Ft increased in the presence of bio-organic fertilizer combined with low amounts of biochar and then slightly decreased in the presence of high amounts of biochar.

The stomatal conductance showed a similar trend as the net photosynthetic rate. In the compound fertilizer group, stomatal conductance decreased in the order B1Fc > B0Fc > B2Fc; additionally, the combination of a low rate of biochar addition enhanced stomatal conductance, while the combination of a high amount of biochar significantly reduced stomatal conductance. In the bio-organic fertilizer group, the highest stomatal conductance was found under B0Ft, after which there was an obvious decrease of 36.7% and 29.6% in the B1Ft and B2Ft treatments, respectively, which indicated that biochar addition had a greater impact on decreasing stomatal conductance when bio-organic fertilizer was applied.

In summary, compared to common fertlizer, the bio-organic fertilizer application increased the transpiration rate, stomatal conductance, and net photosynthesis rate of plants, while reducing the intercellular CO_2_ concentration. Without biochar addition, the photosynthetic characteristics of Chinese small cabbage plants significantly differed from those under compound fertilizer and bio-organic fertilizer application. However, biochar combination hides the difference in photosynthetic characteristics between the two fertilizers, especially at a low biochar addition rate.

### 3.4. Quality of Chinese Small Cabbage

**Figure 8:**
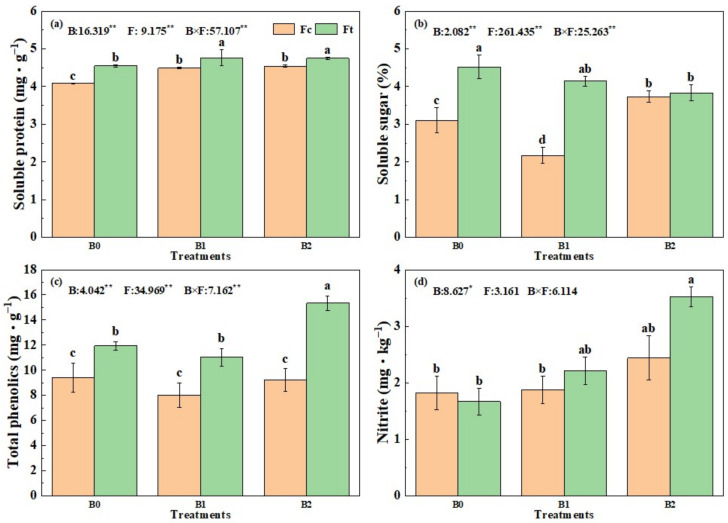
The soluble protein (**a**), soluble sugar (**b**), total phenolic (**c**) and nitrite content (**d**) of Chinese small cabbage under treatments. Notes: “B” represents biochar; B0, B1 and B2 represent the biochar addition rates of 0, 6.85 and 13.7 t·ha^−1^, respectively, with the addition of compound fertilizer (yellow columns) or bio-organic fertilizer (green columns). The different lowercase letters indicate significant difference (at *p* < 0.05) according to Duncan’s test. The values in figures marked after B, F and B × F are the F values of statistics for biochar, fertilizer and their interaction effects, respectively, * and ** indicate significance at the level of 95% and 99%.

As shown in Figure 8, the main effect of biochar, fertilizer and the interactive effect of biochar combined with fertilizer had extremely significant effects on the quality of Chinese small cabbage plants except for the plant nitrite content. These findings indicate that the soluble protein, soluble sugar and total phenolic content were extreme significantly affected by biochar, fertilizer as well as B × F interactions. The effects of B × F interaction on nitrite content were not significant, indicating that nitrite responded similarly, independent of compound fertilizer or bio-organic fertilizer, and that it had a similar response to biochar addition and rate.

The soluble protein content in Chinese small cabbage under B0Fc (4.08 mg·kg^−1^) was significantly lower than the others, which indicated that both bio-organic fertilizer application and biochar addition promoted soluble protein. Notably, the soluble protein content in the B1Fc, B2Fc, B1Ft and B2Ft treatments was significantly greater than those under corresponding treatments without biochar addition (B0Fc and B0Ft), respectively. However, there was no significant difference in the soluble protein content in the biochar addition treatment group.

Generally, the mass fraction of soluble sugars in Chinese small cabbage was greater in bio-organic fertilizer treatment than in the compound fertilizer treatment; in particular, the mass fractions of soluble sugars under B0Ft and B1Ft were significantly greater than those under B0Fc and B1Fc. In the compound fertilizer group, the soluble sugar content decreased significantly with biochar addition and then increased with increasing biochar addition rate, while in the bio-organic fertilizer group, both the biochar addition and its increasing rate continually reduced the mass fraction of soluble sugar in Chinese small cabbage.

The total phenolic content in Chinese small cabbage treated with bio-organic fertilizer was significantly greater than that under the compound fertilizer treatments. Compared to total phenolic under B0Fc (9.41 mg·g^−1^), the total phenolics content in the bio-organic fertilizer group was increased by 17.2% to 63.0%, which indicated that the bio-organic fertilizer application improved the antioxidant capacity of Chinese small cabbage. Interestingly, neither biochar addition nor its rate of increase significantly affected the compound fertilizer group, while a high rate of biochar addition significantly increased the total phenolic content in B2Ft, with a maximum value of 15.34 mg·g^−1^.

Excessive nitrite levels in vegetables pose several threats to human health. The nitrite concentration in Chinese small cabbage under all treatments at harvest was found to be below the national standards (20 mg·kg^−1^) in this trial. With biochar addition and its rate rise, the nitrite content in Chinese small cabbage tended to increase, especially in the bio-organic fertilizer group; the highest nitrite content was 3.52 mg·kg^−1^ under B2Ft, which was near to the limit level. These findings suggest that there is a certain risk of elevated nitrite content under treatments with a high addition of biochar combined with fertilizer, especially bio-organic fertilizer. Notably, the nitrite content was lower in the B0Ft treatment than in the B0Fc treatment, which indicated that the application of single bio-organic fertilizer was safe.

## 4. Discussion

### 4.1. Growth and Yield

Suitable soil conditions, including soil properties, soil water conditions and fertility, are essential for plant growth. In this trial, compared to traditional compound fertilizer without biochar addition, the application of bio-organic fertilizer promoted plant growth, as indicated by the increase in plant height, stem diameter and leaf area index, and significantly enhanced the yield and dry matter accumulation of Chinese small cabbage. These findings are consistent with previous findings from Li that the application of bio-organic fertilizer significantly increased rice yield and yield components [20]. Loh et al. [21] also reported that bio-organic fertilizer provided efficient controlled release and promoted soil microbial rejuvenation and the growth of okra. The research of Duan et al. [22] revealed that fertilization can be a promising method for alleviating water stress, improving soil characteristics, and promoting crop growth. A possible reason is that bio-organic fertilizer has a low bulk density and can reduce soil bulk density and improve soil porosity, and its high organic matter content makes it easier to combine with soil particles and to form stable aggregates. In addition, it is rich in humic acid and mineral nutrients and has long and stable fertility, which is more beneficial for crop growth and uptake. Liu et al. [23] also reported that bio-organic fertilizer promoted the aboveground growth, chlorophyll content and root growth of grape plants and improved the diversity and richness of soil microorganisms.

Notably, the plant growth-promoting effects of biochar combined with fertilizer were greater than those of single fertilizer applications in this trial. On the one hand, biochar addition possibly stimulated and enhanced effects of the fertilizer. It is reported in previous studies that due to the rich porous structure of biochar, it can effectively absorb and store nutrients in fertilizer (with high nutrient retention capacity), reducing nutrient loss and prolonging fertilizer effects [24]. On the other hand, due to its huge specific surface area, biochar can also improve soil structure and provide growth space for soil microorganisms, promote microbial activity, contribute to the decomposition and transformation of organic fertilizer, and release more nutrients for crops to absorb [25]. The plant growth-promoting effects of biochar were also reported for spinach by Wang et al. [26]. Wu et al. [27] revealed that soybean growth and yield were significantly improved by biochar addition.

### 4.2. Accumulated Irrigation Water Amount and Water Productivity

The accumulated irrigation amount under bio-organic fertilizer increased and was much different from that under compound fertilizer in this study, which is consistent with the findings of previous research [28,29]. This could be attributed to the fact that bio-organic fertilizer promoted the photosynthetic rate of plants, as a sharp increase in the accumulated irrigation amount was found at approximately the 20th day after sowing, when the Chinese small cabbage plants were growing vigorously. In addition, an obvious water consumption gap between compound fertilizer and bio-organic fertilizer also occurred at that time. On the other hand, bio-organic fertilizer is rich in organic matter and microflora, and its decomposition process generates heat and enhances the metabolic activities of soil microorganisms, which also consume much water [30]. 

In the bio-organic fertilizer group, the greatest ET, yield and water productivity were obtained under B1Ft, and the peak WP was also observed under B1Fc in the compound fertilizer group, which indicates that there is a threshold of biochar addition and that excessive biochar addition is uneconomical and less effective. A similar conclusion was also reached by Huang [31], who reported that the water use efficiency significantly improved under low and medium biochar addition rates, and there was no help when the addition rate reached 8%. Zhuang [32] also reported that at the same N fertilizer application amount, the dry matter accumulation of plants first increased and then decreased with increasing biochar addition rate, and a moderate increase of 4% was a reasonable amount combined with N fertilizer application. In general, an appropriate rate of biochar addition combined with fertilizer can improve crop yield and water productivity, and we should pay attention to the addition rate of biochar and avoid excess biochar. 

### 4.3. Photosynthesis Characteristics 

Photosynthesis affects the accumulation of organic matter in plants, and the photosynthetic characteristics of Chinese small cabbage plants were improved by bio-organic fertilizer application compared to compound fertilizer without biochar addition in this study. This is in accordance with the findings of Xue et al. [33] and Zhu et al. [34]. This could be explained by that bio-organic fertilizer application improved the soil habitat, balanced the nutrient elements, and led to a synergistic promotion of the physiological and photosynthetic characteristics of plants. Notably, the net photosynthetic rate, transpiration rate and stomatal conductance were reduced by biochar addition in the bio-organic fertilizer group, while a low biochar addition rate combined with compund fertlizer obviously improved the plant photosynthetic characteristics. Comprehensively, these findings indicate that for enhancing plant photosynthesis, it is better to apply compound fertilizer combining biochar addition or to apply bio-organic fertilizer alone. Interestingly, the ET under B1Ft was the highest, while the plant transpiration rate under B1Ft was significantly weaker than that under B0Ft, which suggested that biochar addition was responsible for most of the soil evaporation. Zhan et al. [35] also reported that biochar increased the soil evaporation rate. 

### 4.4. Effects on Plant Quality

Both biochar and bio-organic fertilizer contributed to the improvement in plant quality [36]. In our study, the soluble protein, soluble sugar and total phenolic content of Chinese small cabbage treated with bio-organic fertilizer were greater than those of plants treated with compound fertilzier under the same biochar level. This might be explained by that the bio-organic fertilizer is rich in multiple nutrients, can balance and coordinate biological, inorganic and organic components, and can improve plant growth and the nutritional indices of vegetables. Similarly, Liang [37] reported that bio-organic fertilizer significantly increased the soluble sugar content and total phenol content and decreased the nitrite content in leafy vegetables. It is worth noting that the nitrite content of CSC was mainly affected by biochar addition and waa independent of compound fertilizer or bio-organic fertilizer, which suggests that the application of this kind of bio-organic fertilizer alone is completely safe for vegetable cultivation.

## 5. Conclusions

The growth of Chinese small cabbage was promoted, its yield was enhanced by the application of bio-organic fertilizer with an equal N amount of compound fertilizer, and the effect was equivalent to that of compound fertilizer in this trial. From the perspective of reducing chemical fertilizer application, there is a great possibility to promote the use of such bio-organic fertilizer to replace chemical fertilizer.

The combination of compound fertilizer and biochar effectively promoted plant growth, and the greater the amount of biochar applied, the greater the effect. Considering the needs to save agricultural inputs, the biochar addition rate should not be too high. It is safe and efficient to apply bio-organic fertilizer alone or with lower biochar addition rate; a high rate of biochar addition conversely reduces its growth-promoting effect.

In conclusion, it is considered to be an effective fertilization strategy to combine bio-organic fertilizer with a low amount of biochar to improve land productivity and plant performance in future agriculture in reclaimed land areas based on this trial.

## Figures and Tables

**Table 1:** A detailed description of the treatments.

Treatments	Biochar Addition Rate (t·ha^−1^)	Fertilizer Types
B0Fc (CK)	0	compound fertilizer
B0Ft	0	bio-organic fertilizer
B1Fc	6.85	compound fertilizer
B1Ft	6.85	bio-organic fertilizer
B2Fc	13.7	compound fertilizer
B2Ft	13.7	bio-organic fertilizer

Notes: “B” represents biochar; B0, B1 and B2 represent the biochar addition rate of 0, 6.85 and 13.7 t·ha^−1^; “F” represents fertilizer; Fc and Ft represent the compound fertilizer and bio-organic fertilizer, respectively. For example, “B1Ft” is the treatment of biochar addition at 6.85 t·ha^−1^ combining the bio-organic fertilizer application.

**Table 2:** Yield and WP under treatments.

Treatments	Total Irrigation Water Amount (mm)	ET (mm)	Yield (t·ha^−1^)	WP (kg·m^−3^)
B0Fc	110.36 ± 11.41 c	118.72 ± 7.39 b	23.86 ± 0.96 c	16.45 ± 3.68 c
B1Fc	116.90 ± 10.45 bc	137.51 ± 10.24 ab	28.32 ± 1.21 b	20.51 ± 3.00 b
B2Fc	120.48 ± 16.41 b	143.79 ± 18.99 ab	29.41 ± 5.02 b	20.06 ± 1.25 b
B0Ft	138.78 ± 5.46 ab	152.83 ± 4.57 a	24.81 ± 3.32 c	16.20 ± 1.83 c
B1Ft	141.23 ± 5.18 a	155.86 ± 6.85 a	37.93 ± 4.46 a	26.67 ± 4.43 a
B2Ft	139.40 ± 11.07 ab	151.09 ± 11.86 a	33.67 ± 3.59 ab	22.29 ± 1.74 ab

Notes: “B” represents biochar; B0, B1 and B2 represent biochar addition rates of 0, 6.85 and 13.7 t·ha^−1^, respectively; “F” represents fertilizer; Fc and Ft represent the compound fertilizer and bio-organic fertilizer, respectively. Different lowercase letters indicate significant difference (at *p* < 0.05) in the same column according to Duncan’s test.

## Data Availability

The data that support the findings of this study are available from the corresponding author [J.W.] and [C.L.] upon reasonable request.
